# SPDEF drives pancreatic adenocarcinoma progression via transcriptional upregulation of *S100A16* and activation of the PI3K/AKT signaling pathway

**DOI:** 10.17305/bb.2024.10346

**Published:** 2024-10-01

**Authors:** Hang Jiang, Zhiqian Xue, Liping Zhao, Boyuan Wang, Chenfei Wang, Haihan Song, Jianjun Sun

**Affiliations:** 1 Department of Hepatobiliary and Pancreatic Surgery, The Third People’s Hospital of Yunnan Province, Kunming, Yunnan, China; 2 Shanghai Qibao Dwight High School, Shanghai, China; 3 Department of Emergency, Renji Hospital, School of Medicine, Shanghai Jiao Tong University, Shanghai, China; 4 Department of Immunology, DICAT Biomedical Computation Centre, Vancouver, BC, Canada; 5 Central Lab, Shanghai Key Laboratory of Pathogenic Fungi Medical Testing, Shanghai Pudong New Area People’s Hospital, Shanghai, China

**Keywords:** Pancreatic adenocarcinoma (PAAD), Sam pointed domain-containing ETS transcription factor (SPDEF), *S100A16*, PI3K/AKT signaling pathway

## Abstract

Pancreatic adenocarcinoma (PAAD) is a notably aggressive malignancy with limited treatment options and an unfavorable prognosis for patients. The objective of this study was to elucidate the molecular mechanisms by which Sam pointed domain-containing ETS transcription factor (SPDEF) impacts PAAD progression. Analysis of differentially expressed genes (DEGs) and their association with ETS family members, conducted through The Cancer Genome Atlas (TCGA) database, identified *SPDEF* as a key gene within the molecular framework of PAAD. Kaplan-Meier (KM) survival analysis highlighted the prognostic relevance of SPDEF. In vitro experiments validated the association with cell proliferation and apoptosis, affecting pancreatic cancer cell dynamics. Elevated *SPDEF* expression was observed in PAAD tumor samples, further establishing its role in this disease. Our in vitro assays revealed that *SPDEF* regulates mRNA and protein expression levels, significantly affecting cell proliferation. Moreover, an association was established between SPDEF and a reduction in apoptosis, alongside an increase in cell migration and invasion. An in-depth analysis of *SPDEF*-targeted genes revealed four crucial genes for the advanced prognostic model, among which *S100A16* was significantly correlated with *SPDEF*. Mechanistic analysis showed that *SPDEF* enhances the transcription of *S100A16*, which in turn enhances PAAD cell migration, proliferation, and invasion by activating the PI3K/AKT signaling pathway. Our study revealed the critical role of *SPDEF* in promoting PAAD by upregulating *S100A16* transcription and stimulating the PI3K/AKT signaling pathway, thereby deepening the understanding of the molecular evolution of pancreatic cancer and revealing new therapeutic targets in the SPDEF-driven pathways.

## Introduction

The global prevalence of pancreatic adenocarcinoma (PAAD) has rapidly increased, highlighting the critical importance of early detection and timely intervention [1, 2]. Due to its insidious nature and the lack of distinctive early symptoms, diagnosis is often delayed, resulting in advanced stages of the disease that are frequently inoperable [3, 4]. Late detection of PAAD complicates treatment and reduces the likelihood of a positive outcome for the patient due to the aggressive progression of the cancer. Furthermore, the inherent resistance of PAAD to several conventional treatments means that even if diagnosed early, the treatment effectiveness may still be limited [5]. The genetic heterogeneity of PAAD suggests that tailored treatment options may be more effective than universal strategies [6, 7]. Advancements in personalized medicine and genomic profiling offer promising avenues in this realm. By recognizing the specific genetic aberrations and molecular mechanisms of individual tumors may enable clinicians to design more precise and effective therapeutic interventions.

The PI3K/AKT signaling pathway is crucial for cellular homeostasis and growth. However, when dysregulated, it can contribute to various diseases, particularly cancer [8, 9]. Its role in PAAD is highlighted by a complex network of signaling cascades, feedback mechanisms, and interactions with other pathways, collectively influencing the progression of cancer cells [10, 11]. Aberrant activation of the PI3K/AKT pathway provides a proliferative edge to pancreatic tumor cells [12]. This signaling pathway promotes unchecked cell proliferation, circumvents apoptotic processes, and stimulates angiogenesis, all of which are crucial for tumor survival [13, 14]. Furthermore, it can promote metastasis, which is a primary factor in PAAD fatalities [15]. Recent studies by Stanciu et al. and Li et al. have enriched our understanding of the intricacies of this pathway in pancreatic cancer. Stanciu et al. [16] emphasized the role of cytokines, chemokines, and growth factors in activating the PI3K/AKT/mTOR signaling pathway, revealing the external factors that modulate pancreatic tumor intracellular mechanisms. Conversely, Li et al. [17] demonstrated the therapeutic potential of scoparone, a natural compound, in countering pancreatic cancer by targeting this pathway. Therefore, delving into the nuances of the PI3K/AKT signaling pathway, offers insights into pancreatic cancer mechanisms and promises targeted therapeutic avenues.

The Sam pointed domain-containing ETS transcription factor (SPDEF), while implicated in various cellular processes, has not been extensively explored in the context of PAAD. Unveiling the role of novel molecules, such as *SPDEF*, in the complex molecular landscape of pancreatic cancer may be pivotal for crafting innovative therapeutic avenues. Our investigation focuses on the relationship between *SPDEF* and *S100A16*, its purported downstream effector. Elucidation of this regulatory axis may provide insight into novel molecular mechanisms that may influence the aggressiveness of PAAD. *S100A16*, a member of the S100 protein family known for calcium binding and cellular signaling, may play a nuanced role in tumorigenesis, especially in conjunction with *SPDEF* [18, 19]. Additionally, the potential interplay between *SPDEF*, *S100A16*, and the PI3K/AKT signaling pathway is of considerable interest given the significance of this pathway in various cancers. Such interactions could provide valuable insights into novel avenues of tumor progression and therapeutic resistance. Further investigation into the role of *SPDEF* may reveal its potential as a prognostic marker, providing valuable insights for predicting disease trajectories and guiding treatment decisions in malignancies like PAAD.

In summary, this research aimed to explore the *SPDEF*-*S100A16*-PI3K/AKT axis in the context of PAAD. By elucidating this molecular interaction, the aim is to gain a more complete understanding of the molecular landscape of PAAD and thereby identify potential avenues for targeted therapy, more importantly, hope for patients diagnosed with this formidable malignancy.

## Materials and methods

### Differential gene expression and survival analysis in PAAD

The 179 PAAD samples and four normal samples were obtained from The Cancer Genome Atlas (TCGA) database (https://tcga-data.nci.nih.gov/tcga/). By using the “ggplot2” package in R, differential gene expression analysis was done on the TCGA PAAD dataset. Genes were filtered based on fold change (FC) criteria: FC > 2 or FC < 0.5, and *P* < 0.05 was used as the significance criterion. The distribution of differentially expressed genes (DEGs) was depicted through a volcano plot. The “VennDiagram” package was employed to identify genes that overlapped between DEGs and ETS family genes. The intersection of these gene sets was visualized using Venn diagrams to determine shared candidates for further investigation. The Kaplan-Meier (KM) survival analysis was used to evaluate the prognostic significance of the hub gene (*SPDEF*) concerning overall survival (OS) and recurrence-free survival (RFS) for patients with pancreatic cancer. Survival curves were plotted using “survival” package in R package, with log-rank test *P* values. The relationship between *SPDEF* expression and patient outcomes by comparing high and low-expression groups.

### Expression analysis and staging evaluation of *SPDEF* in PAAD

*SPDEF* expression analysis was performed using the PAAD dataset from the TCGA database. Raw expression data was obtained and processed using R Studio. By using the “ggplot2” package in R, boxplots showing the distribution of *SPDEF* expression in PAAD samples were visualized and *SPDEF* expression levels were evaluated. The relationship between *SPDEF* expression and various pancreatic cancer stages was assessed using the gene expression profiling interactive analysis (GEPIA; http://gepia.cancer-pku.cn/) database. Gene expression data from both the Genotype-Tissue Expression (GTEx) project and TCGA studies were integrated and analyzed by GEPIA. The “Stage Map” function within the GEPIA interface was utilized to investigate SPDEF expression across different tumor stages. Furthermore, the association between *SPDEF* expression with M (metastasis) and N (lymph node) stages was assessed based on clinical data and medical imaging records. Information on metastases and lymph node involvement was collected from clinical reports, imaging studies, and pathological examinations of patients with PAAD. The multifaceted approach, which involved TCGA, GEPIA, and clinical data, enabled a comprehensive analysis of *SPDEF* expression patterns and their potential association with the PAAD tumor stage.

### hTFtarget database

The human transcription factor target gene (hTFtarget, http://bioinfo.life.hust.edu.cn/hTFtarget/) database is specifically designed to predict transcription factor target genes and delineate their regulatory interactions. In this study, the hTFtarget database was utilized to identify genes potentially regulated by *SPDEF*, aiming to uncover *SPDEF* target genes. Subsequently, an intersection analysis was conducted between these predicted genes and the upregulated DEGs (Table S1). This approach provided a more comprehensive understanding of the downstream *SPDEF*-associated regulatory network in the context of PAAD.

### Prognostic analysis of *SPDEF*-related genes in PAAD

The hTFtarget database was used to predict *SPDEF* target genes, which integrates transcription factor-target interactions. A total of thirty genes with significantly altered expression in pancreatic cancer were identified by combining upregulated DEGs and *SPDEF*-predicted target genes. This subset of genes was subjected to the least absolute shrinkage and selection operator (LASSO) regression analysis to create a predictive model. The optimal lambda value (λ_min_ ═ 0.0722) was determined by LASSO regression identifying prognostically significant genes. A prognostic risk model was developed using the expression levels of four significant prognostic genes identified through this analysis process. The risk score formula is as follows: 


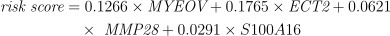


The risk score calculation for pancreatic cancer tumor samples was performed using the TCGA database. Subsequently, samples were dichotomized into high- and low-risk categories based on the median risk score. The performance of the generated risk models was evaluated by survival scatterplots and gene expression heatmaps. The OS was evaluated using KM survival analysis, and the predictive power of the models was evaluated using receiver operating characteristic (ROC) analysis.

### Correlation analysis between *SPDEF* and key prognostic target genes

To explore the potential relationship between *SPDEF* expression and its key prognostic target genes, a correlation analysis was performed. These four key prognostic genes were derived from risk prognostic models. The correlation between *SPDEF* and each target gene was calculated using the Spearman correlation coefficient.

### JASPAR database

The Just Another Spar Promoter Analysis Resource (JASPAR, http://jaspar.genereg.net/) database provides a comprehensive collection of transcription factor binding profiles and matrices. It offers valuable insights into potential binding motifs that transcription factors may recognize within gene promoter regions. In this study, the JASPAR database was used to predict the putative *SPDEF*-binding motif within the promoter region of the *S100A16* gene. This information was critical for elucidating the direct interaction between *SPDEF* and the promoter region of *S100A16*.

### Cell culture

In this study, human normal pancreatic ductal cells (HPNE), as well as the PAAD cell lines (BxPC-3, Capan-2, HPAF-II, PANC-1, MIA PaCa-2, and SW1990) were all sourced from the ATCC (Manassas, VA, USA). Cells were cultured in a humidified incubator at 37 ^∘^C with 5% CO_2_ in a suitable media containing 1% penicillin–streptomycin and 10% fetal bovine serum (FBS). Protein studies were conducted using either the PI3K inhibitor LY294002 or DMSO as a control.

### Cell transfection

PAAD cells were plated at a density of 2 × 10^5^ cells per well in 24-well plates for transient transfection. Plasmids encoding *SPDEF* or *S100A16* were transfected into the PAAD cells to allow for overexpression of these proteins for a designated period. The control group was transfected with vector plasmids. Knockdown was achieved using specific small interfering RNAs (siRNAs) targeting *SPDEF* (si-*SPDEF*#1 and si-*SPDEF*#2) or *S100A16* (si-*S100A16*#1 and si-*S100A16*#2). The knockdown of the control group was performed using non-targeting siRNA, following the manufacturer’s instructions for cell transfection with Lipofectamine 3000 (Invitrogen, USA). Cells were cultured for the optimal amount of time to achieve efficient overexpression or knockdown.

### Quantitative real-time-polymerase chain reaction (qRT-PCR) assay

Total RNA was extracted from PAAD cells using TRIzol (Thermo Fisher Scientific, USA) following the manufacturer’s instructions. cDNA was synthesized using the PrimeScript RT Reagent Kit (Takara, Japan). On a StepOnePlus Real-Time PCR System (Applied Biosystems, USA), qRT-PCR was performed using the SYBR Green PCR Master Mix (Applied Biosystems, USA). The results normalized to an internal standard (glyceraldehyde-3-phosphate dehydrogenase, *GAPDH*). The primer sequences used for amplification were as follows: *SPDEF* forward: 5′-TGTCCGCCTTCTACCTCTCCTAC-3′, *SPDEF* reverse: 5′-CGATGTCCTTGAGCACTTCGC-3′; *S100A16* forward: 5′-GCTGTCGGACACAGGGAAC-3′, *S100A16* reverse: 5′-TGATGCCGCCTATCAAGGTC-3′. The forward and reverse primers for *GAPDH* were as follows: forward: 5′-CAAGCTCATTTCCTGGTATGAC-3′, reverse: 5′-CAGTGAGGGTCTCTCTCTTCCT-3′. The expression was evaluated using the 2^−ΔΔCT^ method.

### Western blotting (WB) assay

Protein lysates from PAAD cells were prepared using RIPA lysis buffer (Thermo Fisher Scientific, USA) containing protease and phosphatase inhibitors. The protein concentration was calculated using the BCA Protein Assay Kit from Thermo Fisher Scientific (USA). Equal quantities of protein were separated using SDS-PAGE and transferred to PVDF membranes from Millipore (USA). The membranes were probed with primary antibodies against Akt (1:1000, Cell Signaling Technology), p-Akt (1:1000, Cell Signaling Technology), p-GSK3β (1:1000, Cell Signaling Technology), SPDEF (1:1000, Abcam), S100A16 (1:1000, Abcam), PI3K (1:1000, Abcam), and GAPDH (1:5000, Cell Signaling Technology) as a control. Following incubation with secondary antibodies, bands were visualized using enhanced chemiluminescence (ECL) and documented with a ChemiDoc imaging system.

### Cell Counting Kit-8 (CCK-8) assay

Cell viability was evaluated using the CCK-8 assay (Dojindo, Japan), on 96-well plates with a seeding density of 5 × 10^3^ PAAD cells per well. Appropriate treatments were administered to each well before adding the CCK-8 reagent. After 0, 24, 48, 72, 96, and 120 h, the absorbance was measured at 450 nm using a microplate reader (Thermo Fisher Scientific, USA).

### Flow cytometry analysis

PAAD cells were separated using Trypsin-EDTA (Gibco, USA) and washed with phosphate-buffered saline. According to the manufacturer’s recommendations, the cells were stained using fluorescently labeled antibodies specific for *SPDEF* and *S100A16* (Abcam, USA). Data were analyzed using FlowJo software (FlowJo LLC, USA) and flow cytometry was carried out using a flow cytometer (BD Biosciences, USA).

### Transwell migration and invasion assay

After 24 hours of transfection, cells were collected and suspended at a density of 5×10^4^ cells/well per well. Subsequently, these cells were loaded into the upper chamber of a six-well Transwell insert. To serve as a chemoattractant, a full medium was added to the lower chamber. After 48 hours of incubation at 37 ^∘^C, non-migratory and non-invasive cells remaining in the upper chamber were meticulously removed with a cotton swab. The cells adhered to the underside of the membrane were fixed using 4% paraformaldehyde and stained with DAPI to visualize nuclei. The invading or migrating cells were observed and quantified using a fluorescent microscope, and images were captured for subsequent analysis.

### PCR analysis of chromatin immunoprecipitation (ChIP)

The ChIP assay was performed using the SimpleChIP Plus Enzymatic Chromatin IP Kit from Cell Signaling Technology and an anti-SPDEF antibody. The DNA fragments were enriched and PCR was performed using primers specific for the predicted *SPDEF* binding motif within the *S100A16* promoter region.

### Luciferase activity assay

A luciferase reporter plasmid with either the wild-type (Wt) or mutant (Mut) *S100A16* promoter sequence was co-transfected into PAAD cells with a plasmid encoding *SPDEF*. The Dual Glo Luciferase Assay System (Promega, Madison, WI, USA) was used following the manufacturer’s instructions to quantify luciferase activity 48 h after co-transfection. Firefly luciferase activity was normalized to the activity of the Renilla luciferase gene.

### Statistical analysis

The Statistical Analysis System was used for all analyses, and experiments were triple-replicated. Data were represented as mean ± SD. Significance (*P* < 0.05) between treatments was ascertained using analysis of variance and Fischer’s test at the 95% confidence level. Mortality differences across treatments were evaluated using a chi-square test.

## Results

### Prognostic significance of *SPDEF* expression in PAAD

**Figure 1.**
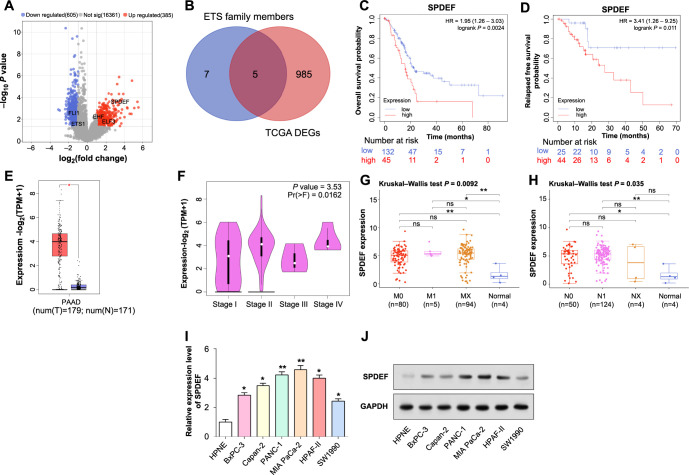
**Expression and prognostic analysis of *SPDEF* in pancreatic PAAD.** (A) Analysis of DEGs in PAAD samples and normal control samples in the TCGA database. Red scattered points represent upregulated DEGs, and blue scattered points represent downregulated DEGs; (B) Venn diagram, analysis of overlapping genes between TCGA-DEGs and ETS family members; (C and D) The impact of differential expression of SPDEF on OS prognosis and RFS prognosis in PAAD patients. Blue represents low-expression samples, red represents high-expression samples; (E) Box plot, validation of *SPDEF* expression in PAAD tumor samples in the GEPIA database; (F-H) Expression of *SPDEF* in different stage subgroups in the GEPIA database, including M stage and N stage; (I and J) qRT-PCR and western blot detected the expression of *SPDEF* in control cells and six PAAD cell lines. *P < 0.05; **P < 0.01. ns: Not significant; PAAD: Pancreatic adenocarcinoma; SPDEF: Sam’s pointed domain-containing ETS transcription factor; DEGs: Differentially expressed genes; TCGA: The Cancer Genome Atlas; GEPIA: Gene expression profiling interactive analysis; qRT-PCR: Quantitative real-time polymerase chain reaction; OS: Overall survival; RFS: Recurrence-free survival.

The TCGA database yielded 385 upregulated and 605 downregulated DEGs between PAAD samples and normal samples (Figure 1A). Further analysis identified five overlapping genes among the DEGs and ETS family members (Figure 1B). To evaluate the prognostic significance of *SPDEF* concerning OS and RFS, KM analysis was conducted. As depicted in Figures 1C and 1D, pancreatic cancer patients with diminished *SPDEF* expression exhibited significantly enhanced OS (*P* ═ 0.0024) and RFS (*P* ═ 0.011). This emphasizes the importance of *SPDEF* as a prognostic determinant and its critical role in disease trajectory and patient prognosis. Further examination revealed a notable increase in *SPDEF* levels in PAAD tumor samples, suggesting its oncogenic function (Figure 1E). Analysis of *SPDEF* expression across PAAD tumor stages identified elevated SPDEF levels in stage 2 tumors (Figure 1F). An in-depth appraisal of both M-stage and N-stage categories corroborated the sustained elevation of *SPDEF* in PAAD tumor specimens, with its expression independent of M-stage and N-stage distinctions (Figures 1G and 1H). For in vitro evaluations, our selection encompassed HPNE and a spectrum of PAAD cells. Through qRT-PCR and WB analytical methodologies, we ascertained a significant upregulation of *SPDEF* in pancreatic cancer cells, predominantly within PANC-1 and MIA PaCa-2 lines (Figures 1I and 1J), designating them for further experimental exploration.

### Effects of *SPDEF* regulation on the phenotype of PAAD cells

**Figure 2.**
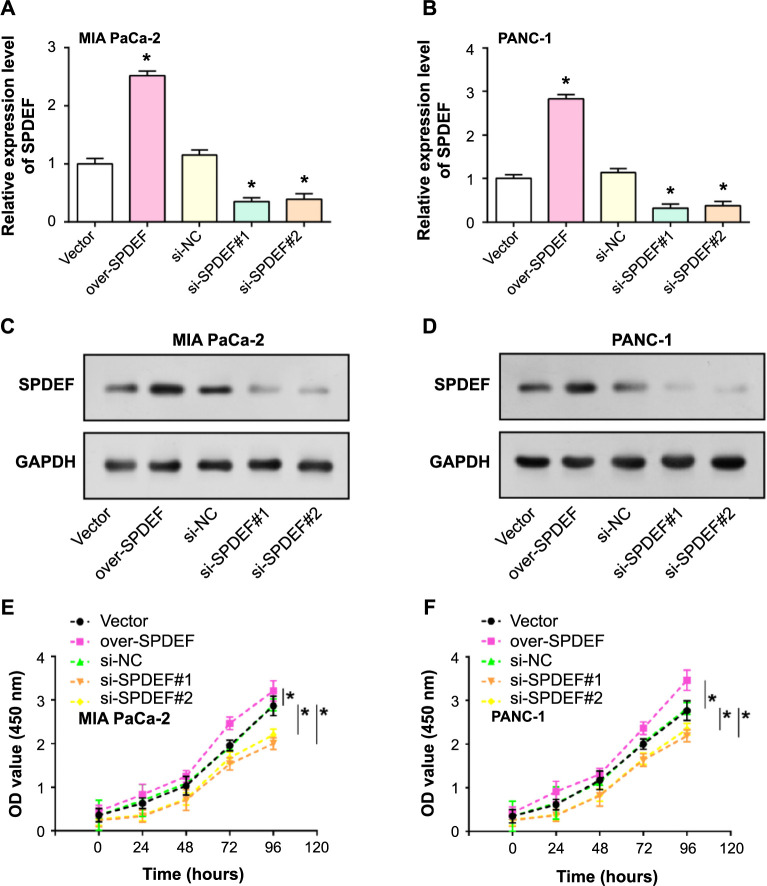
**Regulation of PAAD cell phenotype by overexpression and knockdown of *SPDEF*.** (A-D) qRT-PCR and western blot analyses were used to investigate the effect of *SPDEF* regulation on the phenotype of PAAD cells; (E and F) Functional analysis using the CCK-8 assay to assess cell proliferation in response to SPDEF modulation. *P < 0.05; **P < 0.01. PAAD: Pancreatic adenocarcinoma; SPDEF: Sam pointed domain-containing ETS transcription factor; qRT-PCR: Quantitative real-time polymerase chain reaction; CCK-8: Cell Counting Kit-8.

Utilizing qRT-PCR and WB analyses, we studied the effects of *SPDEF* manipulation on MIA PaCa-2 and PANC-1cells (Figures 2A–2D). Overexpression of *SPDEF* significantly increased both mRNA and protein levels, while *SPDEF* knockdown led to substantial reductions in these levels, with the most pronounced decrease seen in si-*SPDEF*#1. To understand the functional implications of these alterations, the CCK-8 assay was employed. Cells exhibiting increased SPDEF expression demonstrated enhanced proliferation, as indicated by increased absorbance values. However, cells with reduced SPDEF expression displayed diminished proliferation, evidenced by decreased absorbance values (Figures 2E and 2F). Collectively, our data emphasizes the pivotal role *SPDEF* plays in influencing the behavior of PAAD cells.

### SPDEF promotes PAAD cell invasion and metastasis and inhibits apoptosis in vitro

**Figure 3.**
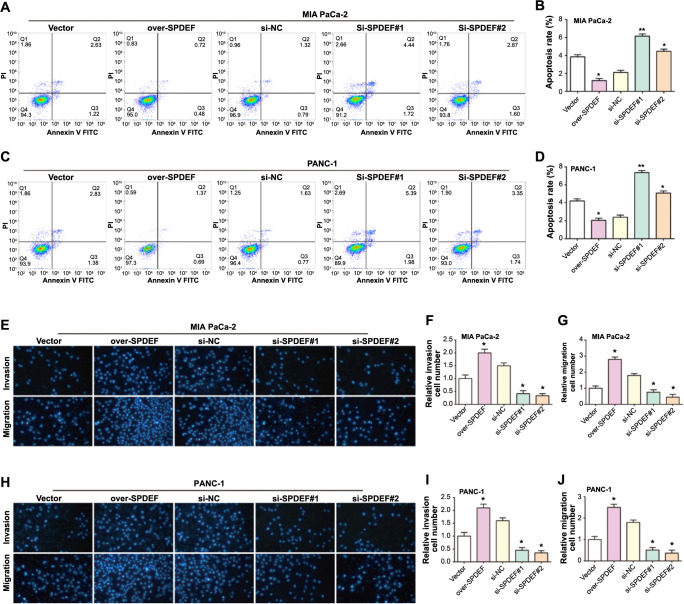
**Differential expression of *SPDEF* regulates PAAD cell apoptosis, migration, and invasion.** (A-D) The effects of *SPDEF* overexpression or knockdown on apoptosis in vitro were evaluated by flow cytometry analysis; (E-J) Transwell experiment to study the effects of *SPDEF* overexpression or knockdown on cell migration and invasion. **P < 0.01. PAAD: Pancreatic adenocarcinoma; SPDEF: Sam pointed domain-containing ETS transcription factor.

Flow cytometry provides a compelling method to study cellular apoptosis. Our results revealed that enhanced SPDEF expression leads to a decline in apoptosis (Figures 3A–3D). Conversely, cells with downregulated SPDEF exhibited pronounced apoptotic activity. To further investigate the effects of SPDEF on PAAD cell dynamics, we used the Transwell assay to analyze cell migration and invasion capabilities. Cells with higher levels of SPDEF exhibited greater migratory and invasive properties. In contrast, cells inhibited with SPDEF demonstrated significantly diminished capacities in both assays (Figures 3E–3J). Together, these insights underscore the instrumental role of SPDEF in shaping the behavior of PAAD cells.

### SPDEF target gene identification and prognostic value analysis

**Figure 4.**
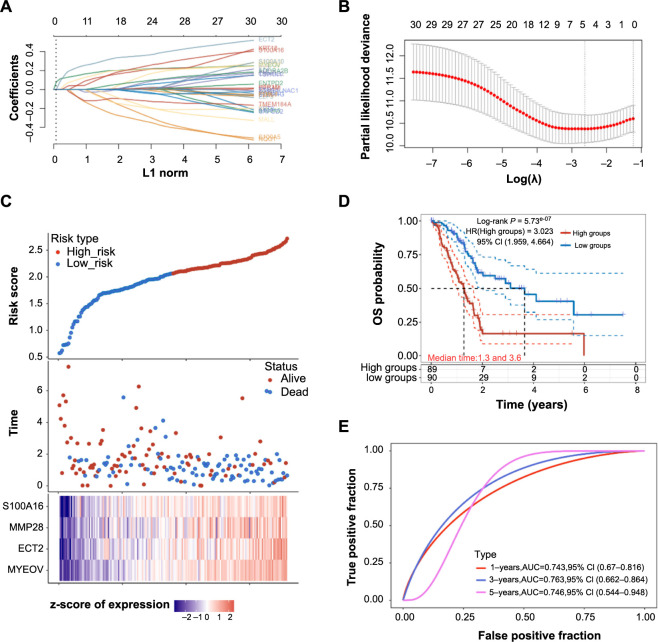
**SPDEF target gene identification and prognostic value analysis in PAAD.** (A) LASSO coefficient profiles of the 30 candidate genes. The vertical line is drawn at the value chosen by 10-fold cross-validation. (B) Partial-likelihood deviance plot versus log(λ). The dotted vertical lines represent the optimal values using the minimum criteria and the one standard error of the minimum criteria. (C) Display of the four significant genes (*S100A16*, *MMP28*, *ECT2*, and *MYEOV*) filtered by LASSO regression, along with their risk scores and survival status. The heatmap below shows the z-score of expression for these genes. (D) Kaplan-Meier survival curves for patients grouped by high and low risk. The number of patients at risk in each group is displayed below the survival curve. (E) ROC curve analysis for the prognostic risk model at 1-, 3-, and 5-year survival periods. AUC values for each interval are provided in the legend. PAAD: Pancreatic adenocarcinoma; SPDEF: Sam pointed domain-containing ETS transcription factor; LASSO: Least absolute shrinkage and selection operator; ROC: Receiver operating characteristic.

The identification of SPDEF target genes is pivotal for deepening our understanding of its function in PAAD. The hTFtarget database was employed to identify these potential targets. An analysis intersecting the predicted targets with upregulated DEGs yielded 30 candidate genes. LASSO regression analysis, with an optimal λ_min_ value pinpointed at 0.0722 (Figures 4A and 4B), further distilled this list to four paramount genes: *S100A16*, *MMP28*, *ECT2*, and *MYEOV* (Figure 4C). Furthermore, KM survival analysis revealed that samples with a higher risk profile have a decreased OS probability (Figure 4D). Additionally, ROC curve analysis indicated that the risk model possessed good prognostic predictive capabilities, with AUC values exceeding 0.7 at the 1-, 3-, and 5-year marks (Figure 4E).

### SPDEF activates transcription of *S100A16*

**Figure 5.**
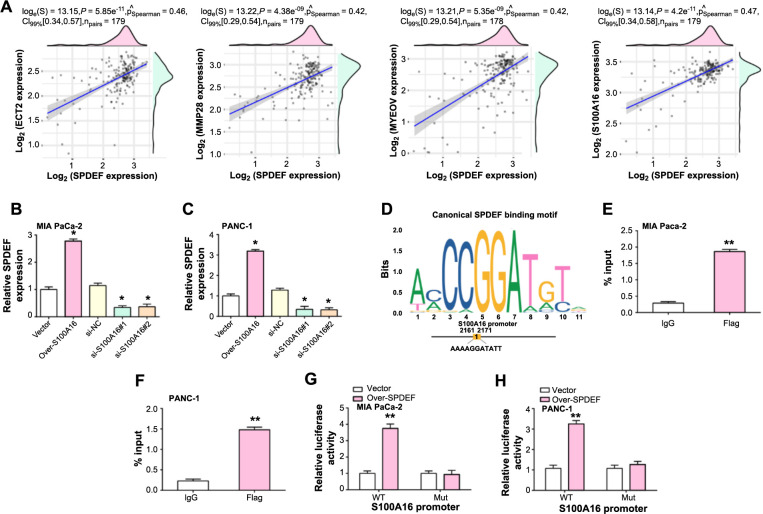
**SPDEF activates the transcription of *S100A16* in PAAD cells.** (A) Correlation analysis between SPDEF expression and its four possible targets (*S100A16*, *MMP28*, *ECT2*, and *MYEOV*). The scatter plots show the correlation coefficient (R) and the *P* value; (B and C) Expression levels of SPDEF after upregulation or knockdown of *S100A16* in MIA PaCa-2 (B) and PANC-1 (C) cells. Bars indicate relative SPDEF expression; (D) The canonical SPDEF binding motif and predicted SPDEF-binding sites within the *S100A16* promoter region; (E and F) ChIP-PCR validation of SPDEF binding to the *S100A16* promoter. The histogram displays enrichment levels of the *S100A16* promoter region in Flag-tagged SPDEF precipitated DNA in MIA PaCa-2 (E) and PANC-1 (F) cells; (G and H) Luciferase reporter assays showing the transcriptional activity of the wild-type and mutated *S100A16* promoter upon SPDEF expression in MIA PaCa-2 (G) and PANC-1 (H) cells. Bars represent relative luciferase activity. *P < 0.05; **P < 0.01. PAAD: Pancreatic adenocarcinoma; SPDEF: Sam pointed domain-containing ETS transcription factor; ChIP: Chromatin immunoprecipitation.

A correlation analysis was conducted involving SPDEF and its four putative targets: *S100A16*, *MMP28*, *ECT2*, and *MYEOV* (Figure 5A). *S100A16* showed the highest correlation with *SPDEF*, prompting us to further investigate its relationship. Experimental observations revealed that elevating *S100A16* levels in PAAD cells led to a surge in *SPDEF* expression (Figures 5B and 5C). Conversely, reducing *S100A16* resulted in decreased *SPDEF* levels. JASPAR was used to predict potential SPDEF-binding sites within the *S100A16* promoter, and several candidates were identified (Figure 5D). To confirm a direct interaction between SPDEF and the *S100A16* promoter, ChIP-PCR was performed. The analysis validated SPDEF enrichment on the *S100A16* promoter, with sequences from the binding region being amplified when DNA was precipitated with Flag-tagged *SPDEF* (Figures 5E and 5F). To further investigate the transcriptional influence of SPDEF on *S100A16*, we introduced both the Wt and the Mut binding site of the *S100A16* promoter into the pGL4.20 vector. After transfection into PAAD cells, luciferase reporter assays showed an increase in *S100A16* promoter activity with SPDEF expression. Notably, this amplification was nullified when the *SPDEF* binding site underwent mutation (Figures 5G and 5H).

### *S100A16* mediates *SPDEF*-induced proliferation, migration, and invasion of PAAD cells

**Figure 6.**
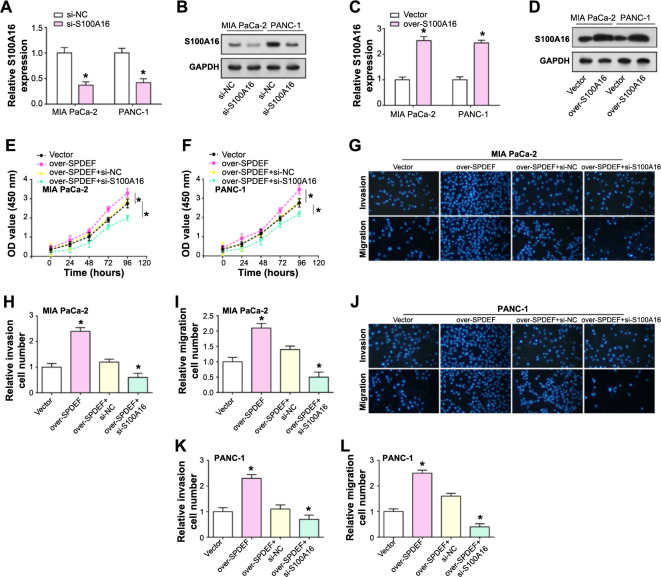
***S100A16* coordinates SPDEF-induced proliferation, migration, and invasion of pancreatic cancer cells.** (A-D) Expression analysis of *S100A16* by qRT-PCR and western blot after *S100A16* knockdown and S00A16 overexpression in PAAD cells; (E and F) CCK-8 assay to determine the regulation of cell proliferation by *S100A16* knockdown and overexpression of SPDEF; (G–L) Transwell assay to evaluate the effects of *S100A16* knockdown and overexpression of SPDEF on cell migration and invasion abilities. *P < 0.05. PAAD: Pancreatic adenocarcinoma; SPDEF: Sam pointed domain-containing ETS transcription factor; qRT-PCR: Quantitative real-time polymerase chain reaction; CCK-8: Cell Counting Kit-8.

A series of experiments were conducted to investigate the interaction between *SPDEF* and *S100A16*, as well as its consequent effects on the behaviors of PAAD cells. qRT-PCR and WB analyses, as illustrated in Figures 6A–6D, demonstrated a significant decrease in *S100A16* expression following its knockdown in PAAD cells. Conversely, the upregulation of *S100A16* was associated with its increased expression. Subsequent functional analyses elucidated the implications of these modulations. The CCK-8 assays showed that the proliferation of PAAD cells increased with heightened *SPDEF* expression. Remarkably, the simultaneous knockdown of *S100A16* in *SPDEF*-overexpressing cells resulted in a significant reduction in proliferation, even greater than the drop observed in the control group (Figures 6E and 6F). To further investigate the role of *S100A16* in *SPDEF*-mediated cellular behaviors, we conducted migration and invasion assays. Figures 6G–6L showed a significant increase in the migratory and invasive potential of PAAD cells under *SPDEF* overexpression. However, a significant finding was observed when *SPDEF*-overexpressing cells were simultaneously subjected to *S100A16* knockdown, as their migration and invasion capabilities were significantly diminished, falling below those of the control group levels.

### SPDEF promotes PAAD progression by transcriptionally upregulating *S100A16* and activating the PI3K/AKT signaling pathway

**Figure 7.**
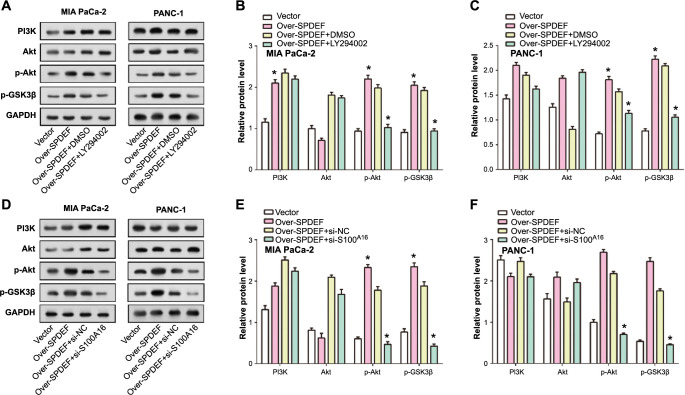
**SPDEF modulates the PI3K/AKT/p-GSK3β signaling cascade in PAAD cells.** (A and B) Western blot analysis representing the impact of SPDEF overexpression, either alone or in conjunction with LY294002 treatment, on the expression levels of PI3K, Akt, p-Akt, and p-GSK3β in MIA PaCa-2 and PANC-1 cells. GAPDH was employed as a loading control; (C) Quantification of protein levels in MIA PaCa-2 and PANC-1 cells treated as described in (A and B); (D and E) Western blot analysis showcasing the effect of SPDEF overexpression either individually or following S100A16 knockdown on the expression of PI3K, Akt, p-Akt, and p-GSK3β in MIA PaCa-2 and PANC-1 cells. GAPDH served as a loading control; (F) Quantitative assessment of protein expressions in MIA PaCa-2 and PANC-1 cells treated as mentioned in (D and E). *P < 0.05. PAAD: Pancreatic adenocarcinoma; SPDEF: Sam pointed domain-containing ETS transcription factor.

To explore the interplay between the PI3K/AKT/p-GSK3β signaling pathway and *S100A16* in cellular dynamics, we conducted a comprehensive experiment using PAAD cells (Figures 7A–7F). Initially, we transfected cells with *SPDEF* overexpression alone, in combination with LY294002 (a PI3K antagonist at 10 µM), or after *S100A16* knockdown at 10 µg/mL. Subsequent WB analyses revealed that SPDEF overexpression led to increased levels of p-Akt, and p-GSK3β. Combining *SPDEF* overexpression with LY294002 resulted in a decline in p-Akt, and p-GSK3β levels, although these levels remained higher than those observed in the control group. Conversely, the combination of *SPDEF* overexpression and S100A16 silencing led to reduced expressions of p-Akt and p-GSK3β. Significant alterations, as measured by WB, were predominantly observed in p-Akt and p-GSK3β. Collectively, our data highlights the pivotal role that S100A16 plays in orchestrating the influence of SPDEF on the PI3K/AKT signaling pathway, thereby illuminating the complex molecular mechanisms that govern the behavior of PAAD cells. 

## Discussion

In this study, we delineate the pivotal influence of the *SPDEF* gene on cancer progression, particularly in the context of the PI3K/AKT signaling pathway. While previous studies have suggested the role of *SPDEF* in various tumors, its unique role in specific cancer manifestations remains unclear. Thus, the multifaceted functions and mechanisms of *SPDEF* in PAAD were deeply investigated. Our data highlighted the integral role of *SPDEF* in the complex progression of PAAD, primarily through the activation of the PI3K/AKT signaling pathway. Although the roles of *SPDEF* and the PI3K/AKT signaling pathway in cancer are recognized, their specific interaction in PAAD remains incompletely explored. The findings of this study help fill this knowledge gap, providing valuable insights into the molecular mechanisms driving PAAD progression.

Initiating our investigation, we employed a bioinformatics approach to identify *SPDEF* as a significant regulator within the complex landscape of pancreatic cancer. *SPDEF* plays a pivotal role in various biological functions [20–22], and while its association with oncogenesis is established, the nuances of its involvement vary across cancer types. For instance, Ye et al. [23] highlighted the dual roles of *SPDEF* in breast cancer, showcasing its oncogenic and tumor-suppressive capacities. Divergently, in colorectal cancers, Lo et al. [22] described that *SPDEF* induces cellular quiescence in colorectal cancer by orchestrating the regulation of β-catenin transcriptional targets. These diverse functions underscore the significant impact of *SPDEF* in cancer biology, marking it as a critical component in developing potential treatments. Our findings indicate a negative correlation between *SPDEF* expression and disease prognosis, with an observed upregulation of *SPDEF* in PAAD tumors, notably in stage 2 tumors. This aligns with the sequential expression pattern in the M and N stages, suggesting a potential proto-oncogene role. Moreover, in vitro functional assessment further revealed the extensive role of *SPDEF* in the regulation of PAAD cells. Overexpression of *SPDEF* resulted in altered cell behavior, promoting proliferation, migration, and invasion while inhibiting apoptosis. In conclusion, our study clarifies the multifaceted role of *SPDEF* in PAAD and offers novel insights into its potential as a diagnostic and therapeutic target.

Through an integrated approach combining bioinformatics analysis, regression techniques, and experimental validation, we precisely identified *S100A16* as a target gene of *SPDEF*, revealing its significant role within the oncogenic landscape. *S100A16*, part of the expansive S100A family, emerges as a key player in cancer biology, exhibiting diverse functions in tumorigenesis [24, 25]. Intriguingly, Li et al. [26] reported the upregulation of *S100A16* and its family members in pancreatic ductal adenocarcinoma (PDAC) tissues compared to normal tissues, a phenomenon inversely related to promoter methylation. This increased expression correlates with diminished survival rates in PDAC patients, highlighting the potential of *S100A16* as a prognostic marker. Notably, the elevated *S100A16* expression in PAAD is intriguingly counteracted by its negative association with immune activity and infiltration, particularly with CD8^+^ T cells. This sheds light on its dual capacity as a prognostic marker and a therapeutic target for immune interventions. Additionally, Li et al. [24] discovered that *S100A16* promotes PDAC metastasis by activating the STAT3 signaling cascade, inducing epithelial-to-mesenchymal transition. This finding suggests that reducing *S100A*16 levels could improve the effectiveness of drugs such as gemcitabine, making it a potential target in the treatment of PDAC. Another study delineated an overexpression of *S100A16* in PDAC, pinpointing its role in advancing the disease through the *FGF19*-mediated AKT and ERK1/2 signaling pathways, further supporting its potential as a therapeutic target [27]. Our findings designate *S100A16* as the primary target gene of *SPDEF* in PAAD, with *SPDEF* expression modulating in response to *S100A16* levels, indicating a direct link between them. Critically, reduced *S100A16* expression mitigated the *SPDEF* overexpression-induced enhancement of PAAD cell proliferation, underscoring its influence on cancer cell dynamics.

The PI3K/AKT signaling pathway is acknowledged as a pivotal regulatory network in exploring the complex mechanisms underlying pancreatic cancer, playing a central role in numerous biological functions [28–30]. This pathway is critical for maintaining healthy cell function but also plays a crucial role in cancer development [31]. Reports suggest that abnormal activation of the PI3K/AKT signaling pathway leads to unlimited proliferation of pancreatic cancer cells and inhibition of apoptosis, supporting tumor growth and metastasis [32]. Another study found that blocking the PI3K/AKT signaling pathway with certain medications can prevent pancreatic cancer cells from migrating and invading, highlighting the critical role of this pathway in pancreatic cancer [33]. Building on this foundation, our study delved into the interaction between the PI3K/AKT/p-GSK3β signaling cascade and *S100A16* in pancreatic cancer cells. Experimental findings revealed that *SPDEF* overexpression led to substantial increases in p-Akt and p-GSK3β levels. These expression levels were reduced when combined with LY294002, the antagonist of PI3K, or *S100A16* knockdown. These results highlight the significant regulatory role of *S100A16* on the PI3K/AKT signaling pathway in modulating the impact of *SPDEF*. The findings shed new light on the molecular basis of pancreatic cancer.

## Conclusion

In summary, our investigation highlights the significant role of *SPDEF* and its target gene, *S100A16*, in the progression and dynamics of PAAD cells. SPDEF was identified as a crucial factor that significantly affects cell proliferation, migration, and invasion. Notably, its transcriptional upregulation of *S100A16* unraveled a significant nexus in shaping PAAD cell behavior. Moreover, the involvement of the PI3K/AKT signaling pathway further elucidates the molecular complexity underpinning the translational effects promoted by *SPDEF* through *S100A16*. These findings shed light on potential therapeutic options and highlight the importance of further investigating these molecular dynamics for effective intervention in PAAD.

## Supplemental data

**Table S1:** List of thirty SPDEF target genes

**Gene**
*C9orf152*
*TMEM184A*
*F2RL1*
*ID1*
*SAPCD2*
*KRT8*
*ENTPD2*
*MALL*
*S100A16*
*EPCAM*
*ECT2*
*MYEOV*
*KRT18*
*ADORA2B*
*HTR1D*
*MANSC1*
*TMC7*
*ST6GALNAC1*
*S100A5*
*ELF3*
*S100A6*
*SH3RF2*
*MMP28*
*NQO1*
*TNS4*
*SEMA4G*
*S100A10*
*TSPAN1*
*F12*
*FOSL1*

## Data Availability

The datasets used and/or analyzed during the current study are available from the corresponding author on reasonable request.
